# Supplementary material for: Studying nucleic envelope and plasma membrane mechanics of eukaryotic cells using confocal reflectance interferometric microscopy
Source: Nat Commun. 2019 Aug 13;10:3652. doi: 10.1038/s41467-019-11645-4 (PMC6692322; doi:10.1038/s41467-019-11645-4)
Supplement: Supplementary file 1 — Supplementary Information [file 41467_2019_11645_MOESM1_ESM.docx]

**Supplementary Information**

**Studying nucleic and plasma membrane mechanics of eukaryotic cells using confocal reflectance interferometric microscopy**

Vijay Raj Singh^1,2,7^, Yi An Yang^3^, Hanry Yu^2,3,4,5^, Roger D. Kamm^2,6,7^, Zahid Yaqoob^1**^ and Peter T. C. So^1,2,6,7^*

***[ptso@mit.edu](mailto:ptso@mit.edu), **[zyaqoob@mit.edu](mailto:zyaqoob@mit.edu)**

*^1^Laser Biomedical Research Center, G. R. Harrison Spectroscopy Laboratory, Massachusetts Institute of Technology, Cambridge, MA 02139, USA*

^2^*Singapore-MIT Alliance for Research and Technology, Singapore 138602*

*^3^Mechanobiology Institute (MBI), National University of Singapore, Singapore 117411*

*^4^Department of Physiology, National University of Singapore, Singapore 117593*

*^5^Institute of Bioengineering and Nanotechnology, Agency for Science, Technology and Research, Singapore 138669*

*^6^Department of Biological Engineering, Massachusetts Institute of Technology, Cambridge, MA 02139, USA*

*^7^Department of Mechanical Engineering, Massachusetts Institute of Technology, Cambridge, MA 02139, USA*

Supplementary Note 1: Confocal reflectance interferometric microscope

**Theoretical framework.** Let the aperture function at DMD-1 plane is{Choi, 2014 #7} a 2D COMB function $C\left( \vec{\mathbf{r}} \right)=\sum_{j_{1},j_{2}} \delta\left( \vec{\mathbf{r}}-j_{1}\Delta\vec{\mathbf{x}}-j_{2}\Delta\vec{\mathbf{y}} \right)$, a rectilinear grid of delta functions corresponding to equally spaced $N\times N$ open apertures with separation between consecutive apertures as $\Delta$. Let ${\vec{\mathbf{r}}}_{s}$ be the raster scan vector coordinate for each grid region defined by the vector $j_{1}\Delta\vec{\mathbf{x}}+j_{2}\Delta\vec{\mathbf{y}}$. In general, ${\vec{\mathbf{r}}}_{s}\in\left[ 0,\vec{\boldsymbol{\Delta}} \right)^{T}.\left[ 0,\vec{\boldsymbol{\Delta}} \right)$ i.e., we only scan within the unit cell defined by the COMB grid. At any given time, the excitation field incident upon the specimen via 4-F relay through the objective is:

$$E_{1}\left( \vec{\mathbf{r}},{\vec{\mathbf{r}}}_{s} \right)=C(\vec{\mathbf{r}}-{\vec{\mathbf{r}}}_{s}) \otimes h\left( \vec{\mathbf{r}} \right)$$

Here, $h\left( \vec{\mathbf{r}} \right)$ is the amplitude point spread function. Let $O\left( \vec{\mathbf{r}} \right)$ be the reflected field by the object for unit illumination, the excitation field back scattered by the object is $E_{2}\left( \vec{\mathbf{r}},{\vec{\mathbf{r}}}_{s} \right)=E_{1}\left( \vec{\mathbf{r}},{\vec{\mathbf{r}}}_{s} \right)O\left( \vec{\mathbf{r}} \right)$. The back scattered field passing back to the DMD-1 plane is $E_{3}\left( \vec{\mathbf{r}},{\vec{\mathbf{r}}}_{s} \right)=E_{2}\left( \vec{\mathbf{r}},{\vec{\mathbf{r}}}_{s} \right)\otimes h\left( \vec{\mathbf{r}} \right)$. The confocal detected field after the DMD-1 can be written as,

$E_{DMD}\left( \vec{\mathbf{r}},{\vec{\mathbf{r}}}_{s} \right)$=$E_{3}\left( \vec{\mathbf{r}},{\vec{\mathbf{r}}}_{s} \right) C\left( \vec{\mathbf{r}}-{\vec{\boldsymbol{r}}}_{s} \right)$

$E_{DMD}\left( \vec{\mathbf{r}},{\vec{\mathbf{r}}}_{s} \right)$=[$\{\left[ C\left( \vec{\mathbf{r}}-{\vec{\mathbf{r}}}_{s} \right)\otimes h\left( \vec{\mathbf{r}} \right) \right]O\left( \vec{\mathbf{r}} \right)\}\otimes h\left( \vec{\mathbf{r}} \right)] C\left( \vec{\mathbf{r}}-{\vec{\mathbf{r}}}_{s} \right)$

$E_{DMD}\left( \vec{\mathbf{r}},{\vec{\mathbf{r}}}_{s} \right)=\sum_{j_{1},j_{2}} \left[ \int d\vec{\mathbf{r}_{\mathbf{2}}}\int d\vec{\mathbf{r}_{\mathbf{1}}}\sum_{i_{1},i_{2}} \delta\left( \vec{\mathbf{r}_{\mathbf{1}}}- {\vec{\mathbf{r}}}_{s}-i_{1}\Delta\vec{\mathbf{x}}-i_{2}\Delta\vec{\mathbf{y}} \right) h\left( \vec{\mathbf{r}_{\mathbf{1}}}-\vec{\mathbf{r}_{\mathbf{2}}} \right)O\left( \vec{\mathbf{r}_{\mathbf{2}}} \right)h\left( \vec{\mathbf{r}_{\mathbf{2}}}-\vec{\mathbf{r}} \right) \right]\delta\left( \vec{\mathbf{r}}- {\vec{\mathbf{r}}}_{s}-j_{1}\Delta\vec{\mathbf{x}}-j_{2}\Delta\vec{\mathbf{y}} \right)$

$E_{DMD}\left( \vec{\mathbf{r}},{\vec{\mathbf{r}}}_{s} \right)$= $\sum_{j_{1},j_{2}} O^{'}(\vec{\mathbf{r}},{\vec{\mathbf{r}}}_{s})\delta(\vec{\mathbf{r}}- {\vec{\mathbf{r}}}_{s}-j_{1}\Delta\vec{\mathbf{x}}-j_{2}\Delta\vec{\mathbf{y}})$ (1)

Here,

$$O^{'}\left( \vec{\mathbf{r}},{\vec{\mathbf{r}}}_{s} \right)= \int d\vec{\mathbf{r}_{\mathbf{2}}}\int d\vec{\mathbf{r}_{\mathbf{1}}}\sum_{i_{1},i_{2}} \delta\left( \vec{\mathbf{r}_{\mathbf{1}}}- {\vec{\mathbf{r}}}_{s}-i_{1}\Delta\vec{\mathbf{x}}-i_{2}\Delta\vec{\mathbf{y}} \right) h\left( \vec{\mathbf{r}_{\mathbf{1}}}-\vec{\mathbf{r}_{\mathbf{2}}} \right)O\left( \vec{\mathbf{r}_{\mathbf{2}}} \right)h\left( \vec{\mathbf{r}_{\mathbf{2}}}-\vec{\mathbf{r}} \right)$$

Electric field corresponding to +1 order, after the common-path interferometer, detected at the camera plane is the object field, which is same as defines in Eq. (1) with phase tilt as,

$E^{+1}\left( \vec{\mathbf{r}},{\vec{\mathbf{r}}}_{s} \right)$= $\sum_{j_{1},j_{2}} O^{'}(\vec{\mathbf{r}},{\vec{\mathbf{r}}}_{s})\delta(\vec{\mathbf{r}}- {\vec{\mathbf{r}}}_{s}-j_{1}\Delta\vec{\mathbf{x}}-j_{2}\Delta\vec{\mathbf{y}})$ $e^{i\vec{\mathbf{k}}\cdot\vec{\mathbf{r}}}$ (2)

The 0^th^ order field is called the reference field, which is generated using the common-path interferometer based on low-pass filtering the object field. Since the excitation pattern is a COMB pattern, thus the Fourier transform of excitation field is also a COMB pattern. To generate the reference field, a COMB aperture filter is used to low pass filter the each comb element at the Fourier plane. The Fourier transform of the excitation field if approximately written as, $\sum_{m} \left( \vec{\mathbf{r}}-{\vec{\mathbf{k}}}_{m} \right)$, which is bunch of plane waves at different directions. Since the field at the Fourier plane multiples the aperture function at DMD-2 plane, the field at the detector convolutes with the Fourier transform of the aperture, give as

$$E^{0}\left( \vec{\mathbf{r}},{\vec{\mathbf{r}}}_{s} \right)= E_{DMD}\left( \vec{\mathbf{r}},{\vec{\mathbf{r}}}_{s} \right)\otimes\sum_{m} e^{i{\vec{\mathbf{k}}}_{m}\cdot\vec{\mathbf{r}}}$$

$$E^{0}\left( \vec{\mathbf{r}},{\vec{\mathbf{r}}}_{s} \right)= \sum_{j_{1},j_{2}} O^{'}(\vec{\mathbf{r}},{\vec{\mathbf{r}}}_{s})\delta(\vec{\mathbf{r}}- {\vec{\mathbf{r}}}_{s}-j_{1}\Delta\vec{\mathbf{x}}-j_{2}\Delta\vec{\mathbf{y}}) \otimes\sum_{m} e^{i{\vec{\mathbf{k}}}_{m}\cdot\vec{\mathbf{r}}}$$

$$E^{0}\left( \vec{\mathbf{r}},{\vec{\mathbf{r}}}_{s} \right)= \int d\vec{\mathbf{r}}\mathbf{'}\sum_{j_{1},j_{2}} O^{'}(\vec{\mathbf{r}}\mathbf{'},{\vec{\mathbf{r}}}_{s})\delta(\vec{\mathbf{r'}}- {\vec{\mathbf{r}}}_{s}-j_{1}\Delta\vec{\mathbf{x}}-j_{2}\Delta\vec{\mathbf{y}}) \sum_{m} e^{i{\vec{\mathbf{k}}}_{m}\cdot\left( \vec{\mathbf{r}^{\mathbf{'}}}-\vec{\mathbf{r}} \right)}$$

$E^{0}\left( \vec{\mathbf{r}},{\vec{\mathbf{r}}}_{s} \right)= \sum_{j_{1},j_{2}} O^{'}({\vec{\mathbf{r}}}_{s}+j_{1}\Delta\vec{\mathbf{x}}+j_{2}\Delta\vec{\mathbf{y}},{\vec{\mathbf{r}}}_{s}) \sum_{m} e^{i{\vec{\mathbf{k}}}_{m}\cdot\left( {\vec{\mathbf{r}}}_{s}+j_{1}\Delta\vec{\mathbf{x}}+j_{2}\Delta\vec{\mathbf{y}}-\vec{\mathbf{r}} \right)}$ (3)

The interference of electric fields of object (Eq. (2)) and reference (Eq. (3)) is detected by the camera and can be written as,

$$I\left( \vec{\mathbf{r}},{\vec{\mathbf{r}}}_{s} \right)=\left( E^{+1}\left( \vec{\mathbf{r}},{\vec{\mathbf{r}}}_{s} \right)+E^{0}\left( \vec{\mathbf{r}},{\vec{\mathbf{r}}}_{s} \right) \right)\times\left( E^{+1}\left( \vec{\mathbf{r}},{\vec{\mathbf{r}}}_{s} \right)+E^{0}\left( \vec{\mathbf{r}},{\vec{\mathbf{r}}}_{s} \right) \right)^{*}$$

$$I\left( \vec{\mathbf{r}},{\vec{\mathbf{r}}}_{s} \right)=E^{+1}\left( \vec{\mathbf{r}},{\vec{\mathbf{r}}}_{s} \right)^{2}+E^{0}\left( \vec{\mathbf{r}},{\vec{\mathbf{r}}}_{s} \right)^{2}+E^{+1}\left( \vec{\mathbf{r}},{\vec{\mathbf{r}}}_{s} \right)E^{0}\left( \vec{\mathbf{r}},{\vec{\mathbf{r}}}_{s} \right)^{*}+E^{+1}\left( \vec{\mathbf{r}},{\vec{\mathbf{r}}}_{s} \right)^{*}E^{0}\left( \vec{\mathbf{r}},{\vec{\mathbf{r}}}_{s} \right)$$

Taking the Hilbert transform of the off-axis interferogram, the final reconstructed field can be written as:

$${T\left( \vec{\mathbf{r}},{\vec{\mathbf{r}}}_{s} \right)= E}^{+1}\left( \vec{\mathbf{r}},{\vec{\mathbf{r}}}_{s} \right)^{*}E^{0}\left( \vec{\mathbf{r}},{\vec{\mathbf{r}}}_{s} \right)$$

$$T\left( \vec{\mathbf{r}},{\vec{\mathbf{r}}}_{s} \right)= \sum_{j_{1},j_{2}} {O^{'}}^{*}(\vec{\mathbf{r}},{\vec{\mathbf{r}}}_{s})\delta(\vec{\mathbf{r}}- {\vec{\mathbf{r}}}_{\mathbf{s}}-j_{1}\Delta\vec{\mathbf{x}}-j_{2}\Delta\vec{\mathbf{y}})\sum_{k_{1},k_{2}} O^{'}({\vec{\mathbf{r}}}_{s}+k_{1}\Delta\vec{\mathbf{x}}+k_{2}\Delta\vec{\mathbf{y}},{\vec{\mathbf{r}}}_{s}) \sum_{m} e^{i{\vec{\mathbf{k}}}_{m}\cdot\left( {\vec{\mathbf{r}}}_{s}+k_{1}\Delta\vec{\mathbf{x}}+k_{2}\Delta\vec{\mathbf{y}}-\vec{\mathbf{r}} \right)}$$

The field at the $j_{1}, j_{2}$ cell can be defined as,

$T\left( j_{1},j_{2},{\vec{\mathbf{r}}}_{s} \right)={O^{'}}^{*}({\vec{\mathbf{r}}}_{s}+j_{1}\Delta\vec{\mathbf{x}}+j_{2}\Delta\vec{\mathbf{y}},{\vec{\mathbf{r}}}_{s})\sum_{k_{1},k_{2}} O^{'}({\vec{\mathbf{r}}}_{s}+k_{1}\Delta\vec{\mathbf{x}}+k_{2}\Delta\vec{\mathbf{y}},{\vec{\mathbf{r}}}_{s})\sum_{m} e^{i{\vec{\mathbf{k}}}_{m}\cdot[\left( k_{1}-j_{1})\Delta\vec{\mathbf{x}}+(k_{2}-j_{2})\Delta\vec{\mathbf{y}} \right)]}$

$T\left( j_{1},j_{2},{\vec{\mathbf{r}}}_{s} \right)= {O^{'}}^{*}(j_{1}\Delta\vec{\mathbf{x}}+j_{2}\Delta\vec{\mathbf{y}}+{\vec{\mathbf{r}}}_{s},{\vec{\mathbf{r}}}_{s})\sum_{k_{1},k_{2}} O^{'}\left( k_{1}\Delta\vec{\mathbf{x}}+k_{2}\Delta\vec{\mathbf{y}}+\vec{\mathbf{r}},{\vec{\mathbf{r}}}_{s} \right)\alpha(j_{1},j_{2},k_{1},k_{2})$ (4)

Here, $\alpha(j_{1},j_{2},k_{1},k_{2})= \sum_{m} e^{i{\vec{\mathbf{k}}}_{m}\cdot[\left( k_{1}-j_{1})\Delta\vec{\mathbf{x}}+(k_{2}-j_{2})\Delta\vec{\mathbf{y}} \right)]}$

Let $O_{R}^{'}$ and $O_{I}^{'}$ be the real and imaginary part of the $O^{'}$, and $\alpha_{R}$ and $\alpha_{I}$are real and imaginary part of $\alpha$. Then Eq. (4) can be simplified as,

$T\left( j_{1},j_{2},{\vec{\mathbf{r}}}_{s} \right)= {[O}_{R}^{'}\left( j_{1},j_{2},{\vec{\mathbf{r}}}_{s} \right)-iO_{I}^{'}\left( j_{1},j_{1,{\vec{\mathbf{r}}}_{s}} \right)]\sum_{k_{1},k_{2}} [O_{R}^{'}\left( k_{1},k_{2},{\vec{\mathbf{r}}}_{s} \right)+{iO}_{I}^{'}\left( k_{1},k_{2},{\vec{\mathbf{r}}}_{s} \right)][\alpha_{R}(j_{1},j_{2},k_{1},k_{2})+i\alpha_{I}(j_{1},j_{2},k_{1},k_{2})]$

Let $T_{R}$ and $T_{I}$are real and imaginary part of $T$. The above equation can be equivalently written as,

$T_{R}\left( j_{1},j_{2},{\vec{\mathbf{r}}}_{s} \right)= \sum_{k_{1},k_{2}} [O_{R}^{'}\left( j_{1},j_{2},{\vec{\mathbf{r}}}_{s} \right){\{O}_{R}^{'}\left( k_{1},k_{2},{\vec{\mathbf{r}}}_{s} \right)\alpha_{R}(j_{1},j_{2,}k_{1},k_{2})-O_{I}^{'}\left( k_{1},k_{2},{\vec{\mathbf{r}}}_{s} \right)\alpha_{I}(j_{1},j_{2},k_{1},k_{2})\}+O_{I}^{'}\left( j_{1},j_{2},{\vec{\mathbf{r}}}_{s} \right)\{O_{R}^{'}\left( k_{1},k_{2},{\vec{\mathbf{r}}}_{s} \right)\alpha_{I}\left( j_{1},j_{2},k_{1},k_{2} \right)+O_{I}^{'}\left( k_{1},k_{2},{\vec{\mathbf{r}}}_{s} \right)\alpha_{R}(j_{1},j_{2},k_{1},k_{2})\}]$ (5a)

$T_{I}\left( j_{1},j_{2},{\vec{\mathbf{r}}}_{s} \right)= \sum_{k_{1},k_{2}} [O_{R}^{'}\left( j_{1},j_{2},{\vec{\mathbf{r}}}_{s} \right){\{O}_{R}^{'}\left( k_{1},k_{2},{\vec{\mathbf{r}}}_{s} \right)\alpha_{I}(j_{1},j_{2},k_{1},k_{2})+O_{I}^{'}\left( k_{1},k_{2},{\vec{\mathbf{r}}}_{s} \right)\alpha_{R}(j_{1},j_{2},k_{1},k_{2})\}-O_{I}^{'}\left( j_{1},j_{2},{\vec{\mathbf{r}}}_{s} \right)\{O_{R}^{'}\left( k_{1},k_{2} \right)\alpha_{R}(j_{1},j_{2},k_{1},k_{2})-O_{I}^{'}\left( k_{1},k_{2},{\vec{\mathbf{r}}}_{s} \right)\alpha_{I}(j_{1},j_{2},k_{1},k_{2})\}]$ (5b)

For each location ${(j}_{1}{,j}_{2})$, there are 2 unknowns ($O_{R}^{'}$ and $O_{I}^{'})$and 2 equations (5(a) and 5(b)). Overall, the system has $2\times N\times N$ unknowns and $2\times N\times N$ equations. A fitting model can be used to unique solutions to these unknown fields.

Quantitative phase reconstruction. As described in Eq. (3), the reference field at each raster scan coordinate is the intensity-weighted sum of unknown specimen field. This results as spatial non-uniformity in reference phase corresponding to each scan position for the field-of-view (FOV) and thus it is specimen dependent. This non-uniformity in reference field phase affects the accuracy of the reconstructed quantitative phase about the specimen. To correct this non-uniformity in the reconstructed phase, the fitting model of non-linear equations, defined in Eq. 5(a)-(b), is used to find the unique solution for object field. After that, a segment in the field-of-view (FOV) called reference segment (scanned by one focus) is chosen corresponding to the flat region of the specimen to correct the phase jumps at other foci scan positions in field-of-view. The phase value of the first scan position is measured at foci in reference segment. The phase differences of remaining scan positions of foci are measured from the phase of first scan of the reference segment. These phase difference values at corresponding scan positions are subtracted for all foci of remaining segments. Since the phase of reference segment is same at all scan coordinates, this subtraction process for the entire scanning steps corrects the spatial phase variations of the reference field caused by the intensity-weighted sum of specimen field.

**
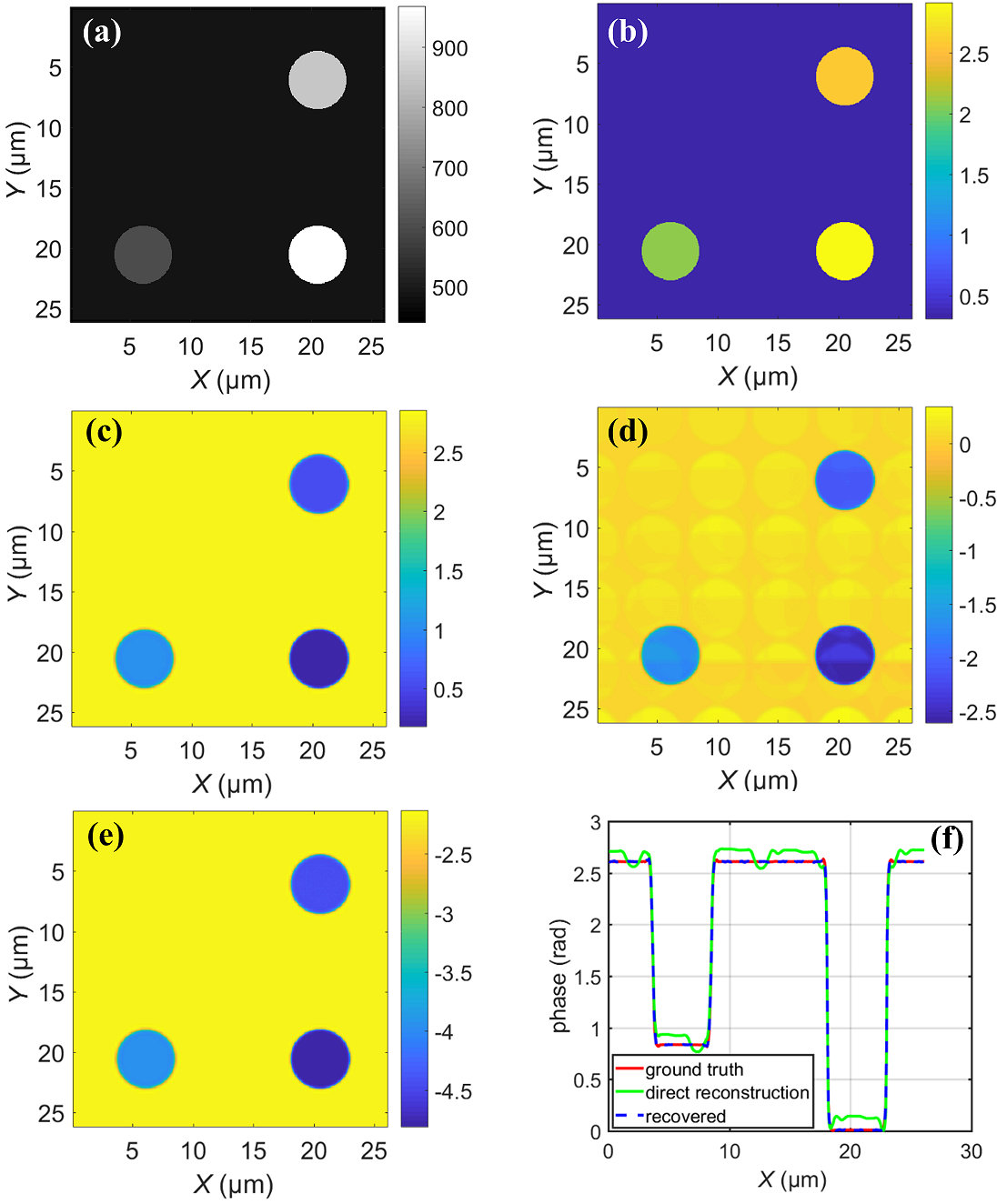
**

**Supplementary Figure 1:** Simulation results showing correct phase reconstitution. (a) and (b) Simulated object amplitude and phase, respectively. (c) Phase of the field detected before interferometer (ground truth). (d) Phase of the field reconstructed using the interferometer. (e) Recovered phase of the field using the proposed algorithm. (f) Line plot comparing the phase of the fields quantitatively.

**Simulation results.** The model presented in section S1 is tested with the simulations and results are shown in Supplementary Fig. 1. Complex object is simulated as spatially varying amplitude and phase information as shown in Supplementary Fig. 1(a) and (b). The phase of the field detected after DMD-1, as shown in Supplementary Fig. 1(c), is considered as the ground truth that need to be recovered. When the common path interferometer is used, the intensity weighted sum of the specimen field creates artifacts in the reference field and it results as artifacts in the direct reconstructed field. Such artifacts can be clearly seen in the reconstructed phase, as shown in Supplementary Fig. 1(d).

To correct such artifacts, the algorithm presented earlier is used to recover phase image as shown in Supplementary Fig S(e). Clearly, the recovery of the phase is accurate and quantitative comparison is shown in Supplementary Fig. 1(f). For fitting the non-linear equation as discussed in Eq. 5(a) and 5(b), we utilize the Matlab® function ‘*fsolve*’ while considering direct reconstructed field as the initial guess. For the simulation, camera well depth is considered at 30K e^-^. The spatial noise of the recovered phase is calculated as 3.5mrad, which is below the shot noise limit of the camera (5.8mrad) [1].

**Supplementary Note 2:**  **Calibration of confocal reflectance interferometric microscope**

Supplementary Figure 2 shows the basic characterization for the system. Supplementary Figure 2(a) shows the axial point spread function - plot of reconstructed intensity as a function of axial position. Next, the phase noise of the system is evaluated by recording a temporal sequence of interferograms for a static glass-water interface. For each interferogram, the well depth of the camera is filled by keeping the counts of bright fringes close to the saturation level of the detector pixel (well depth is 30,000 electrons). Next, instantaneous phase variation maps are computed by subtracting the mean phase map from the sequence of phase maps, i.e., $\Delta\phi\left( x,y;t \right)=\phi\left( x,y,t \right)-\left\langle\phi\left( x,y \right) \right\rangle$. Supplementary Figure 2(b) shows a plot of the reconstructed phase fluctuations $\Delta\phi$ at a single spatial position, called the phase noise plot. Finally, a reflective positive US air force (USAF) resolution target is used as a phase specimen, where resolution bars are fabricated using lithography process, and the step height of these resolution bars is 100nm. Supplementary Figure 2(c) shows the interferogram recorded when the group 7 element region is placed in focus. The reconstructed phase representing specimen’s topography, after converted phase valued to the height map as shown in Supplementary Fig. 2(d). The step height is measured by plotting the line profile along the group element number 7 as shown in the inset. This shows that phase reconstruction accurately reconstruct the 3D height measurement of specimen.

**
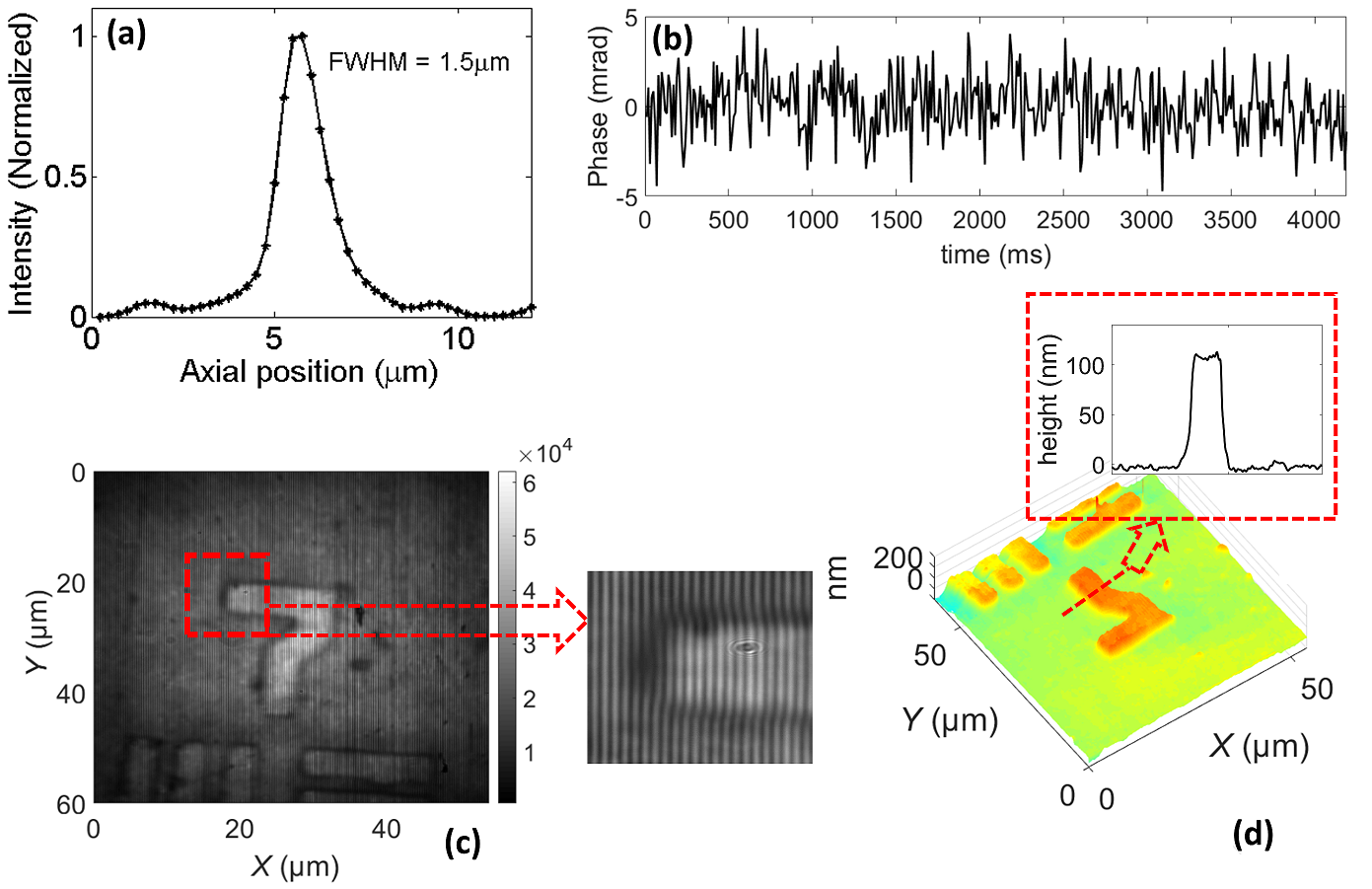
**

**Supplementary Figure 2:** Calibration results of the system. (a) Axial point spread function. (b) Phase noise of the system plotted as a function of time for the glass-water interface. (c) Recorded interferogram of the positive USAF target; inset is zoomed region showing the interference fringes around the coated pattern. (d) 3D reconstructed map from the measured interferogram. The phase is converted into the height map; inset shows the line profile of the region indicated by dotted red line.

**Supplementary Note 3:**  **Accurate displacement measurement of membrane fluctuations in the presence of glass interface reflection**

In the proposed multi-point scanning confocal phase microscope, the reference field is generated by the specular reflection from a highly reflective coverglass interface, located at an off-focal plane, underneath the cell. This approach provides a highly phase-stabilized reference, which is critical for desired phase measurement stability. However, the presence of strong signal from the glass interface also suppresses the measured phase associated with the fluctuating membrane. Previous literature suggests that the presence of strong reflector, in close proximity to the interface of interest, leads to suppressed phase measurement in low-coherence depth-resolved optical imaging systems [2]. We note that the approach presented in Ref [3] is based on point-illumination, where several measurements are needed to account for phase leakage. We developed a systematic approach to recover the accurate phase from light contribution from different interfaces using a single wide-field interferogram. In the following, we describe the theoretical model, simulations, and experimental results presenting accurate measurement of membrane fluctuations in healthy red blood cells (RBCs). For validation, we also measured RBC membrane fluctuations using two additional systems, namely, diffraction phase microscope (DPM) [3] and dynamic speckle illumination phase microscope [4].

Let $E_{c}$ and $E_{0}$ are the amplitudes of signals from in-focus cellular membrane and off-focus glass interface respectively. $\phi_{0}$ and $\phi_{c}^{0}$ represent the mean phase associated with light arriving from glass interface and the cell membrane. Furthermore, $\phi\left( t \right)$ is the temporal phase fluctuations of the membrane under observation; glass interface is assume to be static. The total back scattered field, $E_{T}$, can be written as,

$E_{T}=E_{0}e^{i\phi_{0}}+E_{c}e^{i\{\phi_{c}^{0}+\phi\left( t \right)\}}$ (6)

Supplementary Figure 3 shows the plot of rms phase of the total backscattered field (red trace) as a function of contribution from glass interface. Clearly, the phase is accurate when there is no contribution from glass interface. On the other hand, the phase fluctuation is suppressed when signal from glass contributes to the total field; however, the amount of suppression is deterministic. A common-path interferometer is placed in the detection arm of the system to evaluate the phase of the total field. The interference of object and reference fields is recorded at the camera plane, where the object field is the same as the total field as defined in Eq. 6. Finally, the reference field is generated from the total field using a customized filter. For our near-common path system, the generation of reference field at any location on the camera is the spatial average of the total field detected from all the foci in the field-of-view (Eq. 3). Thus for the total object field, defined in Eq. 6, the reference field generated at the camera in our system can be defined as $E_{R}={\bar{E_{o}}e}^{i\bar{\phi_{o}}}+{\bar{E_{c}}e}^{i\bar{\phi_{c}}}$, where $\bar{E_{o}}$ and $\bar{E_{c}}$ are the spatially averaged amplitudes of optical signal contributions from the glass and the membrane interfaces, respectively. Further, $\bar{\phi_{o}}$ and $\bar{\phi_{c}}$ are their corresponding spatially averaged phase shifts. Since $\bar{E_{c}}$ depends on the fractional area covered by the cells in the field-of-view (FOV), the generation of reference field is completely governed by the cell membrane contributions in absence of signal from glass interface. In such a scenario, the variation in cell density within the FOV will lead to a non-stable reference field. For instance, rms phase of fluctuating membrane is not recovered at all for 10% or less cell density for in the absence glass contribution (see the first data point of yellow trace in Supplementary Fig. 3). However, as the glass contribution increases, the rms phase of the total field starts to recover. For larger than 10% cell densities, rms phase can be measured without any glass contribution, but not without significant error. Thus, a stable reference field from the glass interface, with similar or larger amplitude than that of the fluctuating membrane, is advantageous for the recovery of accurate phase fluctuation information. In our experiments, the contribution of glass interface (normalized by the membrane signal) is above 2, which is essential for stable phase recovery.


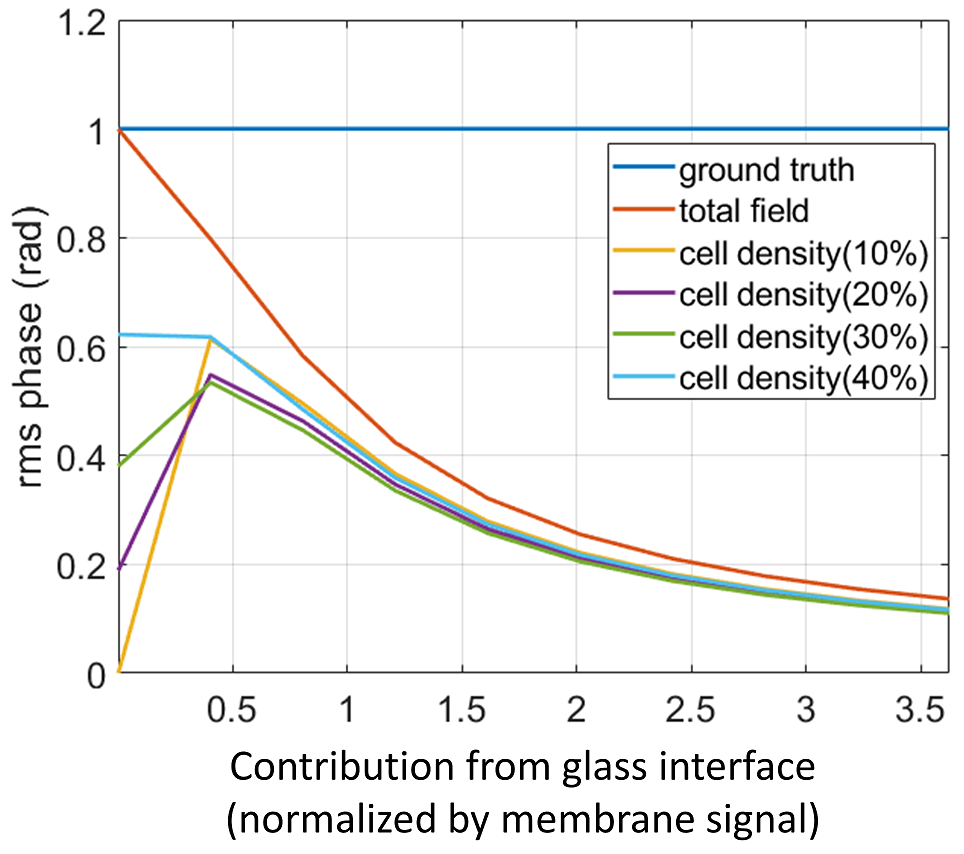


**Supplementary Figure 3:** Suppression in measured phase associated with total field as a function of optical signal from the fluctuating membrane and contribution from the glass interface.

As discussed earlier, while the glass interface signal ensures a stable reference field, its static nature also results in “phase suppression”. In the following, we describe a model for correcting the suppressed phase. For off-axis interferometry, the reference field can be defined as,

$E_{R}=E_{r}e^{i\phi_{r}}e^{ik_{x}}$, (7)

where $e^{ik_{x}}$ is the off-axis phase tilt. The detected interferogram can be written as

$$I={(E}_{T}+ E_{R})\times{{(E}_{T}+ E_{R})}^{*}$$

$I=E_{0}^{2}+E_{r}^{2}+E_{c}^{2}+{2E}_{0}E_{c}\cos\left( \phi_{0}-\phi_{c}^{0}-\phi\left( t \right) \right)+2E_{r}E_{o}cos(\phi_{r}+k_{x}-\phi_{0})+{2E}_{r}E_{c}cos(k_{x}+\phi_{r}-\phi_{c}^{0}-\phi\left( t \right))$ (8)

Next, the interferogram is processed after taking its Fourier transform:

$$\tilde{I}=\left[ E_{0}^{2}+E_{r}^{2}+E_{c}^{2}+{2E}_{0}E_{c}\cos\left( \phi_{0}-\phi_{c}^{0}-\phi\left( t \right) \right) \right]\delta\left( k_{x} \right)+E_{r}E_{o}(\delta\left( k{+k}_{x} \right)+\delta\left( k{-k}_{x} \right))e^{i({\phi_{0}-\phi}_{r})}+E_{r}E_{c}\left( \delta\left( k{+k}_{x} \right)+\delta\left( k{-k}_{x} \right) \right)e^{i({\phi_{c}^{0}+\phi\left( t \right)-\phi}_{r})}$$

The phase directly reconstructed from off axis interferogram, using Hilbert transform, can be written as,

$\varphi=\tan^{-1} (\frac{E_{r}E_{o}sin({\phi_{0}-\phi}_{r})+E_{r}E_{c}\sin\left( {\phi_{c}^{0}+\phi\left( t \right)-\phi}_{r} \right)}{E_{r}E_{o}cos({\phi_{0}-\phi}_{r})+E_{r}E_{c}\cos\left( {\phi_{c}^{0}+\phi\left( t \right)-\phi}_{r} \right)})$ (9)

As discussed before, the direct phase recovered from interferogram is suppressed due to presence of signal from glass interface, i.e. $E_{o}$.

To recover the corrected phase, the DC and AC component of the interferogram can be written as:

$I_{DC}=E_{0}^{2}+E_{r}^{2}+E_{c}^{2}+{2E}_{0}E_{c}cos(\phi_{0}-\phi_{c}^{0}-\phi\left( t \right))$ (10)

$I_{AC}=E_{r}E_{o}e^{i({\phi_{0}-\phi}_{r})}+E_{r}E_{c}e^{i({\phi_{c}^{0}+\phi\left( t \right)-\phi}_{r})}$ (11)

For outside the cell region, magnitude of the DC is $E_{0}^{2}+E_{r}^{2}$ and magnitude of the AC is $E_{r}E_{o}$. By using the measured DC and AC values and solving the algebraic equations, one can evaluate the values of $E_{r}$ and $E_{c}$.

Finally, by rearranging the AC term, the recovered phase can be calculated as

$\phi_{r}-\phi_{c}^{0}-\phi\left( t \right)=\tan^{-1} (\frac{Im(I_{AC})-E_{r}E_{o}\sin\left( \phi_{r}-\phi_{0} \right)}{Re(I_{AC})-E_{r}E_{o}cos(\phi_{r}-\phi_{0})})$ (12)

where, $Re(I_{AC})$ and $Im(I_{AC})$ are the real and imaginary part of AC, inside the cells region.

Membrane fluctuations of healthy red blood cell (RBC) measured for experimental validation. The phase of the membrane is converted to the height followed by the RMS fluctuations measurements that are further compared with the one measured using the diffraction phase microscopy (DPM) [3] and dynamic speckle phase microscopy system [4], for the same RBCs population. DPM system is a well-known transmission-type wide-field interferometric tool for measuring nanometer scale thermally driven RBCs membrane fluctuations that is also near-common path [5, 6]. On the other hand, dynamic speckle phase microscope is a recently developed wide-field reflection-type interferometric tool that provides depth-resolved quantitative phase maps of biological samples similar to the current manuscript although it is less sensitivity due to not being common path. It has been shown to selectively measure top membrane fluctuations in RBCs [4]. We believe that comparing the RBC membrane fluctuations, measured using the proposed system, with the above-mentioned two modalities should provide a fair assessment of accuracy of the system.

The blood sample was washed two times with PBS and centrifuged at 821 × g at 21 °C for 5 min. Following two washes with PBS, fractionated RBCs were suspended in PBS with 1% BSA (Sigma-Aldrich) where 1 μL of pellet was suspended in 200 μL of PBS–BSA and kept at 4 °C until use. Alsever’s Solution from Sigma-Aldrich is use as an isotonic solution for RBCs while measuring the membrane fluctuations.

First, we record a series of interferograms using the proposed system by keeping the focal plane at top membrane plane of a RBC. Supplementary Figure 4(a) shows the phase image of a RBC directly reconstructed from the measured interferogram whereas Supplementary Fig. 4(b) and (c) illustrate the corresponding RMS fluctuation and instant displacement maps. We observe a typical value of ~0.15 radian rms phase, which corresponds to ~5 nm membrane fluctuation amplitude. This value is significantly lower than that measured with DPM (~35 nm) or dynamic speckle phase microscope (~21 nm). We note that higher rms fluctuation amplitude is observed with DPM (than dynamic speckle) due to its transmission geometry, which records fluctuations of both top and bottom membranes of the RBC. Next, we use the proposed reconstruction model to compute the phase maps as well as the instantaneous displacement and rms fluctuation maps, as shown in Supplementary Figs. 4(d-f). As expected, the recovered rms phase fluctuations was improved and measured to be ~0.6 radians. An improvement of ~4 times in rms phase is also expected since the amplitude of signal from glass interface was measured about 1 to 2 times higher than the amplitude of signal from the membrane, which points to a phase suppression of 3-4 times (Supplementary Fig. 3). A comparison of original and corrected temporal phase fluctuations measured at a single spatial location is also shown in Supplementary Fig. 4(g). Further, the measurements were extended to a number of RBCs (n = 7) using the proposed confocal reflectance interferometric system. For comparison, the measurements of membrane fluctuations of the same RBC population were also performed using the dynamic speckle phase microscopy and DPM systems. More precisely, the rms fluctuation amplitude was measured at 4 different spatial locations on each RBC. As shown in Supplementary Fig. 4(h), the rms fluctuations measured using the proposed system are comparable with that measured using dynamic speckle phase system. This is due to the fact that both the systems essentially provide depth-resolved phase measurement in back scattered mode. We note that the rms fluctuation measured using dynamic speckle based system is slightly higher, which may be attributed to higher phase noise because of its non-common path geometry. Further, approximately $\sqrt{2}$ higher rms fluctuation amplitude is expected for DPM due to its transmission geometry, which measures fluctuation contributions from both top and bottom membranes.

**
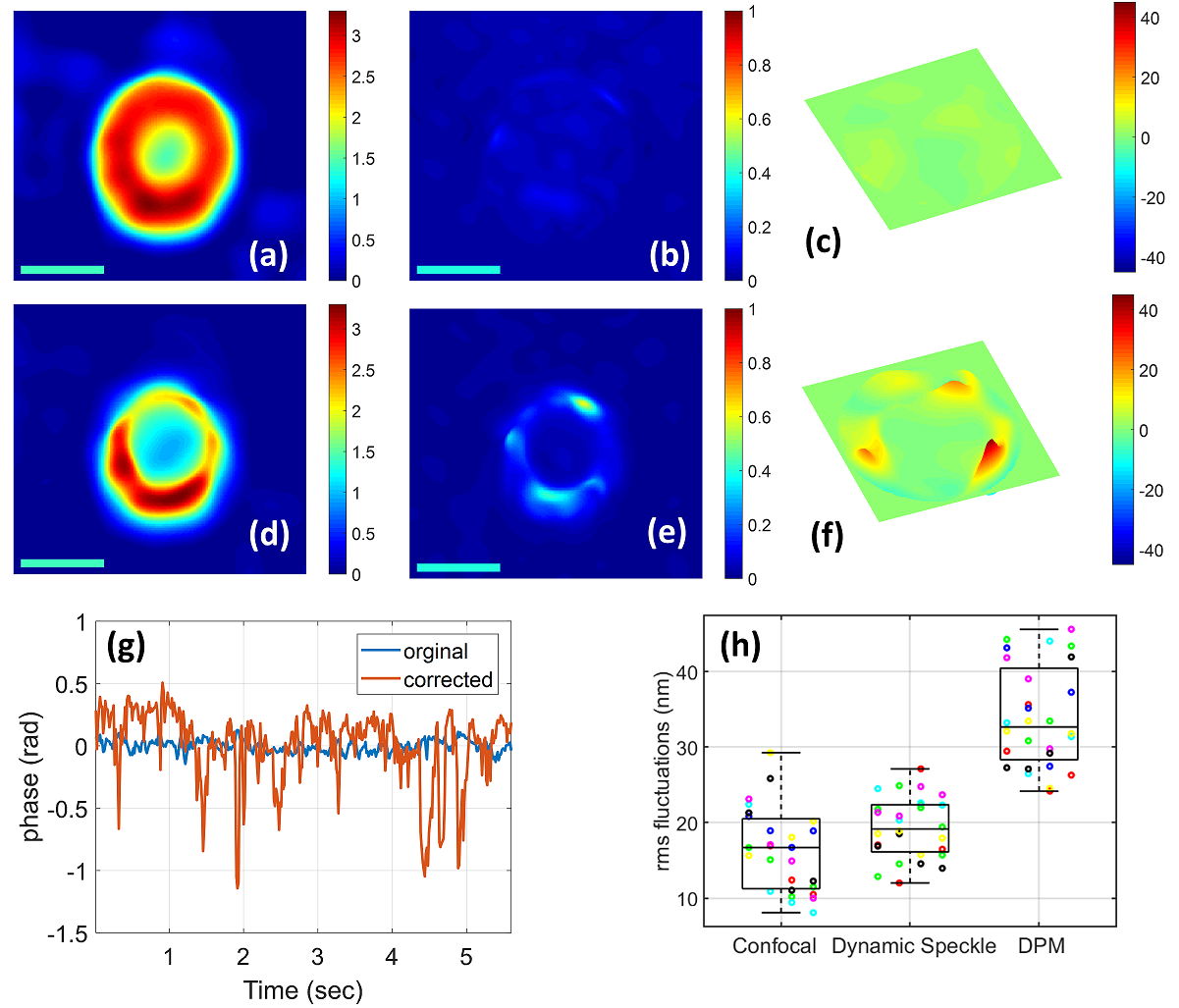
**

**Supplementary Figure 4:** Recovery of suppressed phase for red blood cell membrane fluctuations. (a)-(c) are phase image of RBC, its rms phase fluctuation map and instantaneous height map (scale bar is in nm) corresponding to original phase reconstruction. (d)-(f) are the corresponding (as shown in (a)-(c)) corrected phase image, rms phase fluctuations and instantaneous height map, respectively, by using the proposed method (scale bar 5µm). (g) Comparison of original and corrected temporal phase of membrane, and (h) Comparison of RBCs membrane rms fluctuations measured from presented confocal interferometric microscopy, dynamic speckle based phase and DPM systems. The line within each box represents the median, and the lower and upper boundaries of the box indicate the first and third quartiles, respectively. Error bars (whiskers) represent the interquartile range (*n* = 28).

**Supplementary Note 4: Nucleic and membrane fluctuations animation**

Supplementary Figure 5 shows the animation video of membrane fluctuations of cell bottom interface, nucleic and plasma membrane fluctuations of embryonic stem cells. The phase reconstructed from the temporal sequence of recorded interferograms recorded corresponding to nucleic and plasma membranes is converted to the height map [7, 8]. The topography plot sequence, reconstructed at each time point, is showing the corresponding membrane fluctuations.


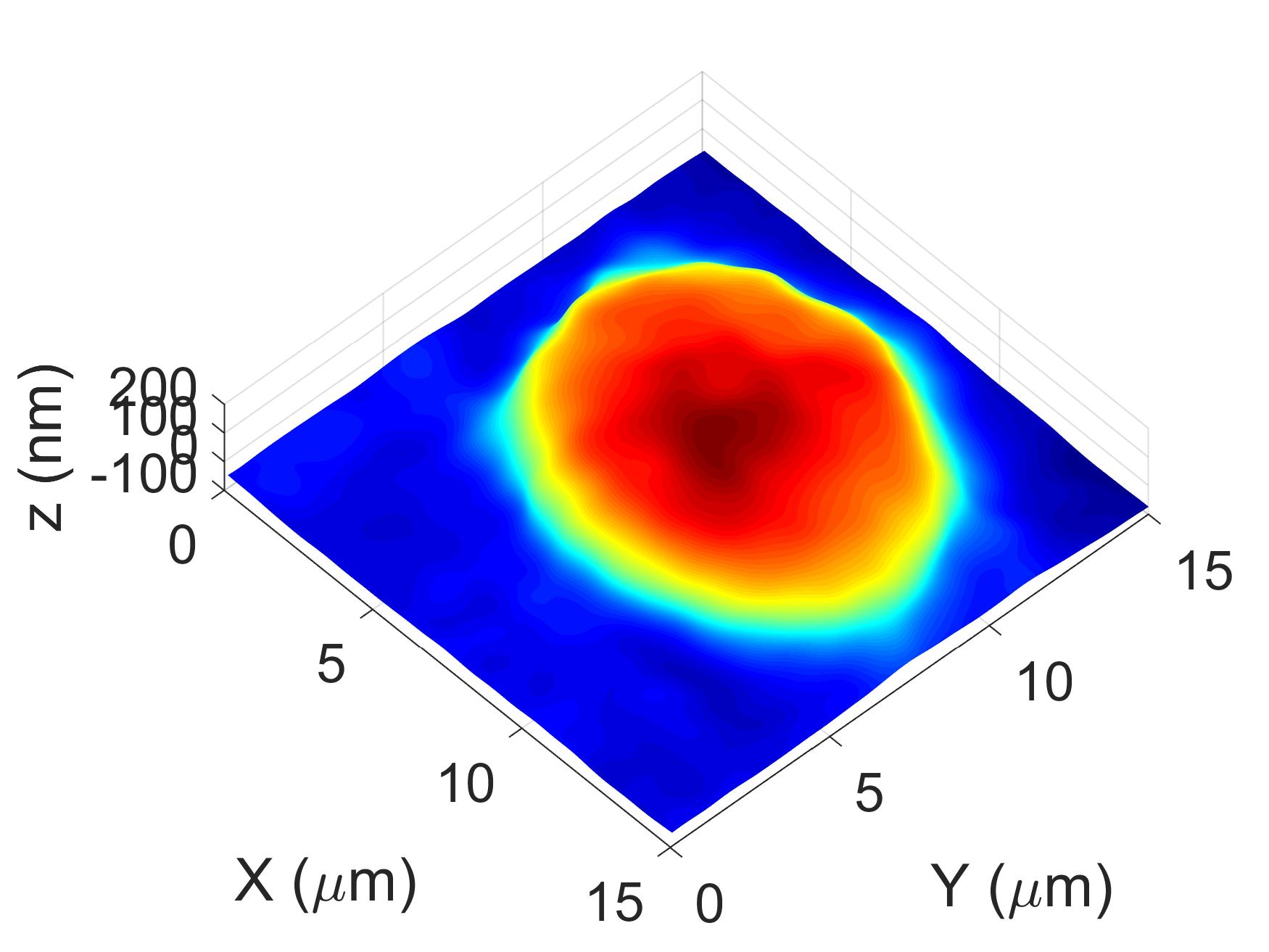

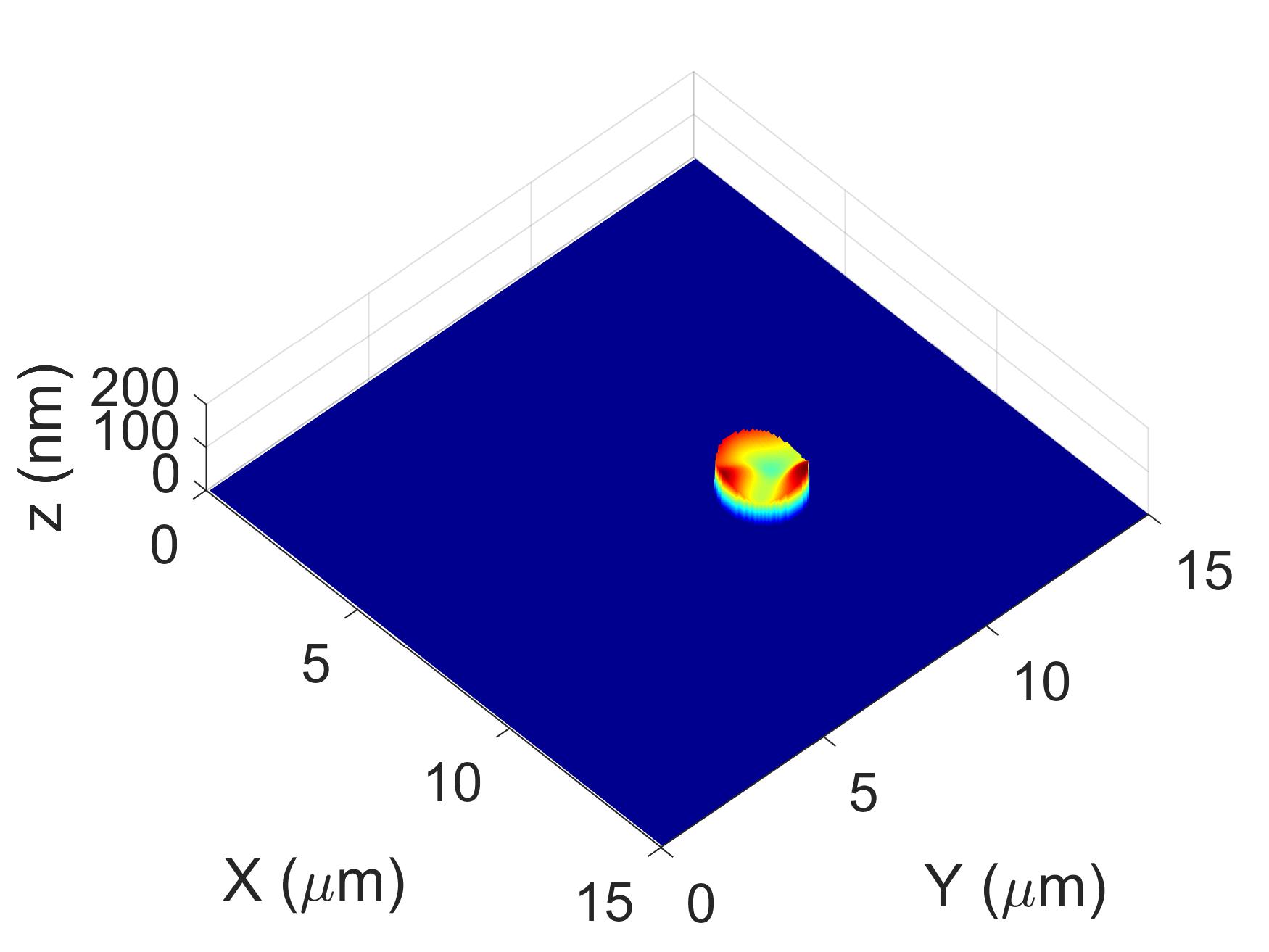

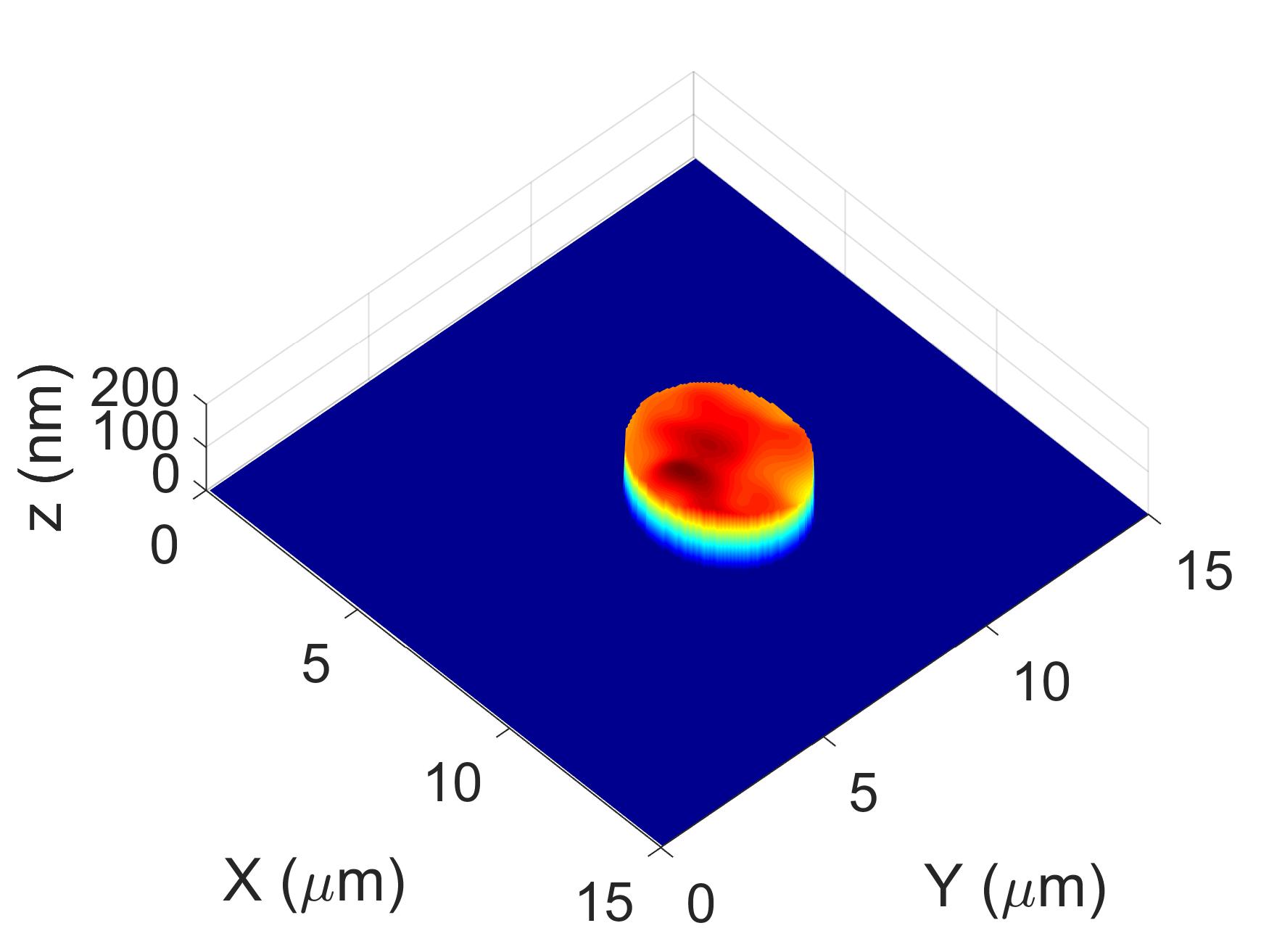


***(a) (b) (c)***

**Supplementary Figure 5:** Animations of membrane fluctuations of embryonic stem cells. (a) Bottom region of the cell, (b) Nucleic membrane fluctuations, and (c) Plasma membrane fluctuations.

**Supplementary Note 5:**  **Optical sectioning plot for different pinholes size and their separations**

Supplementary Figure 6 shows the plot of axial resolution of the system for different pinhole size and their separation. Supplementary Figure 6(a) shows the decay of axial resolution while increasing the size of the pin-hole. The plot shows the average intensity for recorded image as a function of axial scan position. The FWHM obtained from the plots are $1.5 \mu m$ and $2.0 \mu m$ corresponding to the pinhole size $1.03 \mu m$ and $1.37 \mu m$ respectively. We note that the separation of pinholes is kept about 10 times the size of each pinhole. Reducing the separation of pinholes requires less the number of scanning patterns and thus increases the imaging speed; it, however, degrades the depth resolution. The separation between the pinholes are kept as $10 \mu m$ for both plots. Supplementary Figure 6(b) shows the degradation of axial plot when the separation of the pinholes is reduced. For this plot, the pinhole size are kept constant as $1.03 \mu m$ and their separation is reduced. Clearly, the reduction in pinhole separation results as poor sectioning and additional side lobes appears, which degrade the performance of the system.


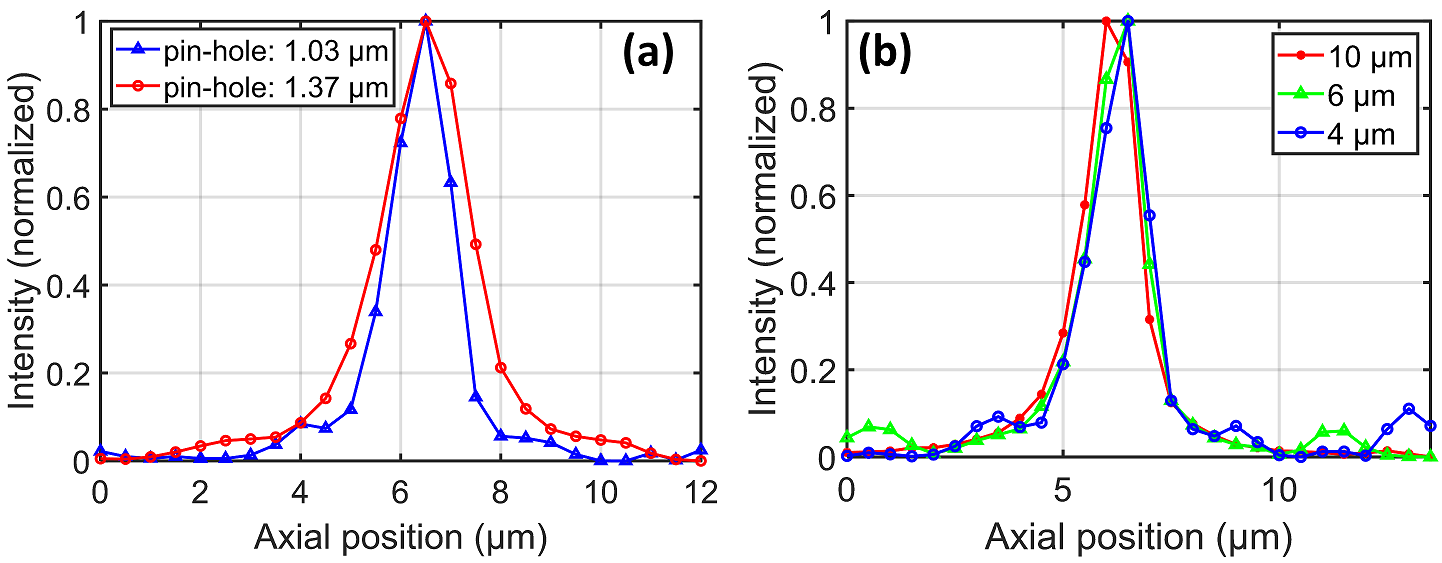


**Supplementary Figure 6:** Plots of axial point spread function of the system corresponding to (a) two different pinhole sizes and (b) different separation of the pinholes.

**Supplementary References**

1. P. [Hosseini](https://www.ncbi.nlm.nih.gov/pubmed/?term=Hosseini%20P%5BAuthor%5D&cauthor=true&cauthor_uid=27192311), R. [Zhou](https://www.ncbi.nlm.nih.gov/pubmed/?term=Zhou%20R%5BAuthor%5D&cauthor=true&cauthor_uid=27192311), Y.H. [Kim](https://www.ncbi.nlm.nih.gov/pubmed/?term=Kim%20YH%5BAuthor%5D&cauthor=true&cauthor_uid=27192311), C. [Peres](https://www.ncbi.nlm.nih.gov/pubmed/?term=Peres%20C%5BAuthor%5D&cauthor=true&cauthor_uid=27192311), A. [Diaspro](https://www.ncbi.nlm.nih.gov/pubmed/?term=Diaspro%20A%5BAuthor%5D&cauthor=true&cauthor_uid=27192311), C. [Kuang](https://www.ncbi.nlm.nih.gov/pubmed/?term=Kuang%20C%5BAuthor%5D&cauthor=true&cauthor_uid=27192311), Z. [Yaqoob](https://www.ncbi.nlm.nih.gov/pubmed/?term=Yaqoob%20Z%5BAuthor%5D&cauthor=true&cauthor_uid=27192311), and [P.T. So](https://www.ncbi.nlm.nih.gov/pubmed/?term=So%20PT%5BAuthor%5D&cauthor=true&cauthor_uid=27192311), “Pushing phase and amplitude sensitivity limits in interferometric microscopy”, *Opt. Lett*., 41 (7), 1656, 2016.
2. A.K. Ellerbee, and J.A. Izatt, “Phase retrieval in low-coherence interferometric microscopy”, *Opt. Lett*., Vol. 32, 388-390 (2007).
3. G. Popescu, T. Ikeda, R.R. Dasari, and M.S. Feld, “Diffraction phase microscopy for quantifying cell structure and dynamics”*.* *Opt. Lett.*, Vol. 31: p. 775-777 (2006).
4. Y. Choi, P. Hosseini, W. Choi, R.R. Dasari, P.T. So, and Z. Yaqoob, “Dynamic speckle illumination wide-field reflection phase microscopy”, *Opt. Lett.*, Vol. 39, 6062 (2015).
5. Y.K. Park, C. Depeursinge, and G. Popescu, “Quantitative phase imaging in biomedicine”, *Nat. Photonics*, Vol. 12, 578-589 (2018).
6. [T. Ling](https://www.nature.com/articles/s41377-018-0107-9#auth-1), [K.C. Boyle](https://www.nature.com/articles/s41377-018-0107-9#auth-2), [G. Goetz](https://www.nature.com/articles/s41377-018-0107-9#auth-3), [P. Zhou](https://www.nature.com/articles/s41377-018-0107-9#auth-4), [Y. Quan](https://www.nature.com/articles/s41377-018-0107-9#auth-5), [F.S. Alfonso](https://www.nature.com/articles/s41377-018-0107-9#auth-6), [T.W. Huang](https://www.nature.com/articles/s41377-018-0107-9#auth-7), and [D. Palanker](https://www.nature.com/articles/s41377-018-0107-9#auth-8), “Full-field interferometric imaging of propagating action potentials”, *Light: Science & Applications*, 7: 107 (2018).
7. P. Groot, “Principles of interference microscopy for measurement of surface topography”, *Adv. Opt. and Photonics,* Vol. 7, 1 (2015).
8. C. Hu, and G. Popescu, “Physical significance of backscattereing phase measurements”, *Opt. Lett.*, Vol. 42, 4643 (2017).
