# Supplementary material for: Studying nucleic envelope and plasma membrane mechanics of eukaryotic cells using confocal reflectance interferometric microscopy
Source: Nat Commun. 2019 Aug 13;10:3652. doi: 10.1038/s41467-019-11645-4 (PMC6692322; doi:10.1038/s41467-019-11645-4)
Supplement: Supplementary file 3 — Description of Additional Supplementary Files [file 41467_2019_11645_MOESM3_ESM.pdf]

Description of Additional Supplementary Files

File Name: Supplementary Movie 1

Description: Bottom region of the cell.

File Name: Supplementary Movie 2

Description: Nucleic envelope fluctuations.

File Name: Supplementary Movie 3

Description: Plasma membrane fluctuations.
