# Supplementary material for: Studying nucleic envelope and plasma membrane mechanics of eukaryotic cells using confocal reflectance interferometric microscopy
Source: Nat Commun. 2019 Aug 13;10:3652. doi: 10.1038/s41467-019-11645-4 (PMC6692322; doi:10.1038/s41467-019-11645-4)
Supplement: Supplementary file 7 — Reporting Summary [file 41467_2019_11645_MOESM7_ESM.pdf]

nature research

Corresponding author(s): NCOMMS-18-14762A

Last updated by author(s): Jul 19, 2019

Reporting Summary

Nature Research wishes to improve the reproducibility of the work that we publish. This form provides structure for consistency and transparency in reporting. For further information on Nature Research policies, see Authors & Referees and the Editorial Policy Checklist.

Statistics

For all statistical analyses, confirm that the following items are present in the figure legend, table legend, main text, or Methods section.

n/a	Confirmed	
☐	☒	The exact sample size (*n*) for each experimental group/condition, given as a discrete number and unit of measurement
☐	☒	A statement on whether measurements were taken from distinct samples or whether the same sample was measured repeatedly
☐	☒	The statistical test(s) used AND whether they are one- or two-sided<br>*Only common tests should be described solely by name; describe more complex techniques in the Methods section.*
☐	☒	A description of all covariates tested
☐	☒	A description of any assumptions or corrections, such as tests of normality and adjustment for multiple comparisons
☐	☒	A full description of the statistical parameters including central tendency (e.g. means) or other basic estimates (e.g. regression coefficient) AND variation (e.g. standard deviation) or associated estimates of uncertainty (e.g. confidence intervals)
☒	☐	For null hypothesis testing, the test statistic (e.g. *F*, *t*, *r*) with confidence intervals, effect sizes, degrees of freedom and *P* value noted<br>*Give P values as exact values whenever suitable.*
☒	☐	For Bayesian analysis, information on the choice of priors and Markov chain Monte Carlo settings
☒	☐	For hierarchical and complex designs, identification of the appropriate level for tests and full reporting of outcomes
☒	☐	Estimates of effect sizes (e.g. Cohen's *d*, Pearson's *r*), indicating how they were calculated

Our web collection on statistics for biologists contains articles on many of the points above.

Software and code

Policy information about availability of computer code

Data collection	Camera software and MATLAB software were used to collect the data from the camera integrated with the hardware.
Data analysis	MATLAB software was used to perform the data analysis.

For manuscripts utilizing custom algorithms or software that are central to the research but not yet described in published literature, software must be made available to editors/reviewers. We strongly encourage code deposition in a community repository (e.g. GitHub). See the Nature Research guidelines for submitting code & software for further information.

Data

Policy information about availability of data

All manuscripts must include a data availability statement. This statement should provide the following information, where applicable:

- Accession codes, unique identifiers, or web links for publicly available datasets
- A list of figures that have associated raw data
- A description of any restrictions on data availability

The interferometric data and corresponding codes that support the findings of this study are available on request from the corresponding author.

Field-specific reporting

Please select the one below that is the best fit for your research. If you are not sure, read the appropriate sections before making your selection.

☒ Life sciences ☐ Behavioural & social sciences ☐ Ecological, evolutionary & environmental sciences

For a reference copy of the document with all sections, see nature.com/documents/nr-reporting-summary-flat.pdf

Life sciences study design

All studies must disclose on these points even when the disclosure is negative.

Sample size	Undifferentiated mouse embryonic stem cells were seeded, on a 35mm petri dish, at a moderate density (10^3 cells cm^-2) in culture medium without leukemia inhibitory factor. It provides few cells for measurement in the field-of-view of the system.
Data exclusions	No data were excluded from the analysis.
Replication	Experiment were repeated with multiple embryonic stem cells and analyzed results highlighting cell to cell variability.
Randomization	For a given interface (nucleic envelope or plasma membrane), the region of interest (ROI) is selected in the middle of the interface. For analysis, the data points are chosen randomly from the selected ROI.
Blinding	Blinding is not relevant for our study as the proposed instrument is tested with known cell types with known substrate properties.

Reporting for specific materials, systems and methods

We require information from authors about some types of materials, experimental systems and methods used in many studies. Here, indicate whether each material, system or method listed is relevant to your study. If you are not sure if a list item applies to your research, read the appropriate section before selecting a response.

Materials & experimental systems

n/a	Involved in the study
☒	☐ Antibodies
☐	☒ Eukaryotic cell lines
☒	☐ Palaeontology
☒	☐ Animals and other organisms
☒	☐ Human research participants
☒	☐ Clinical data

Methods

n/a	Involved in the study
☒	☐ ChIP-seq
☒	☐ Flow cytometry
☒	☐ MRI-based neuroimaging

Eukaryotic cell lines

Policy information about cell lines

Cell line source(s)	Mouse embryonic stem cells E14TG2a (ATCC, CRL-1821).
Authentication	Cells were routinely examined by their morphology in subculture and immunostaining of pluripotency marker Oct4.
Mycoplasma contamination	We did not perform mycoplasma screening for the cell line used.
Commonly misidentified lines (See ICLAC register)	No commonly misidentified cell lines were used in this study.
